# 1571. Hospital COVID-19 Burden Impact on Inpatient Antibiotic Use Rates

**DOI:** 10.1093/ofid/ofac492.100

**Published:** 2022-12-15

**Authors:** Elizabeth Dodds Ashley, Yuliya Lokhnygina, Danielle Doughman, Katherine R Foy, Alicia D Nelson, April Dyer, Travis M Jones, Melissa D Johnson, Angelina Davis, Sonali D Advani, Andrea Cromer, Nikolaos Mavrogiorgos, Lindsay M Daniels, Ashley H Marx, Ibukun Kalu, Emily Sickbert-Bennett, S Shaefer Spires, Deverick J Anderson, Rebekah W Moehring

**Affiliations:** Duke Center for Antimicrobial Stewardship and Infection Prevention, Durham, North Carolina; Duke University, Durham, North Carolina; University of North Carolina Medical Center, Chapel Hill, North Carolina; Duke Center for Antimicrobial Stewardship and Infection Prevention, Durham, North Carolina; Duke University Medical Center, Durham, North Carolina; Duke Center for Antimicrobial Stewardship and Infection Prevention, Durham, North Carolina; Duke Center for Antimicrobial Stewardship and Infection Prevention, Durham, North Carolina; Duke University School of Medicine, Durham, North Carolina; Duke Center for Antimicrobial Stewardship and Infection Prevention, Durham, North Carolina; Duke University School of Medicine, Durham, North Carolina; Duke Center for Antimicrobial Stewardship and Infection Prevention, Durham, North Carolina; University of North Carolina Medical Center, Chapel Hill, North Carolina; UNC Medical Center, Chapel Hill, North Carolina; University of North Carolina Medical Center, Chapel Hill, North Carolina; Duke University, Durham, North Carolina; UNC Medical Center, Chapel Hill, North Carolina; Duke University, Durham, North Carolina; Duke University, Durham, North Carolina; Duke University, Durham, North Carolina

## Abstract

**Background:**

COVID-19 shifted antibiotic stewardship program resources and changed antibiotic use (AU). Shifts in patient populations with COVID surges, including pauses to surgical procedures, and dynamic practice changes makes temporal associations difficult to interpret. Our analysis aimed to address the impact of COVID on AU after adjusting for other practice shifts.

**Methods:**

We performed a longitudinal analysis of AU data from 30 Southeast US hospitals. Three pandemic phases (1: 3/20–6/20; 2: 7/20–10/20; 3: 11/20–2/21) were compared to baseline (1/2018–1/2020). AU (days of therapy (DOT)/1000 patient days (PD)) was collected for all antimicrobial agents and specific subgroups: broad spectrum (NHSN group for hospital-onset infections), CAP (ceftriaxone, azithromycin, levofloxacin, moxifloxacin, and doxycycline), and antifungal. Monthly COVID burden was defined as all PD attributed to a COVID admission. We fit negative binomial GEE models to AU including phase and interaction terms between COVID burden and phase to test the hypothesis that AU changes during the phases were related to COVID burden. Models included adjustment for Charlson comorbidity, surgical volume, time since 12/2017 and seasonality.

**Results:**

Observed AU rates by subgroup varied over time; peaks were observed for different subgroups during distinct pandemic phases (Figure). Compared to baseline, we observed a significant increase in overall, broad spectrum, and CAP groups during phase 1 (Table). In phase 2, overall and CAP AU was significantly higher than baseline, but in phase 3, AU was similar to baseline. These phase changes were separate from effects of COVID burden, except in phase 1 where we observed significant effects on antifungal (increased) and CAP (decreased) AU (Table).

**Conclusion:**

Changes in hospital AU observed during early phases of the COVID pandemic appeared unrelated to COVID burden and may have been due to indirect pandemic effects (e.g., case mix, healthcare resource shifts). By pandemic phase 3, these disruptive effects were not as apparent, potentially related to shifts in non-COVID patient populations or ASP resources, availability of COVID treatments, or increased learning, diagnostic certainty, and provider comfort with avoiding antibacterials in patients with suspected COVID over time.

**Disclosures:**

**Melissa D. Johnson, PharmD**, Biomeme: Licensed Transcriptional Signature for Candidemia|Charles River Laboratories: Grant/Research Support|Entasis Therapeutics: Advisor/Consultant|Merck & Co. Inc: Advisor/Consultant|Merck & Co. Inc: Grant/Research Support|Pfizer, Inc.: Advisor/Consultant|Scynexis Inc.: Grant/Research Support|Theratechnologies: Advisor/Consultant **Angelina Davis, PharmD, M.S.**, Merck & Co.: Honoraria **Sonali D. Advani, MBBS, MPH, FIDSA**, Locus Biosciences: Advisor/Consultant|Locus Biosciences: Honoraria|Sysmex America: Advisor/Consultant **Ibukun Kalu, MD**, Pfizer, Inc.: Institutional support for clinical trial **Rebekah W. Moehring, MD, MPH, FIDSA, FSHEA**, UpToDate, Inc.: Author Royalties.

